# Clinical Utility of Duplex Ultrasonography in the Recognition of Transplant Renal Artery Stenosis: A Single Center Experience

**DOI:** 10.3390/diagnostics15141766

**Published:** 2025-07-13

**Authors:** Ahmad Mirza, Usman Baig, Munazza Khan, Shameem Beigh, Imran Gani

**Affiliations:** 1 Transplant Surgery Division, Wellstar MCG Health, Augusta University, Augusta, GA 30912, USA; 2 Department of Nephrology, Hypertension and Transplant Medicine, Wellstar MCG Health, Augusta University, Augusta, GA 30912, USAsbeigh@augusta.edu (S.B.); igani@augusta.edu (I.G.); 3 Medical University—Pleven, 1, Saint Kliment Ohridski Street, 5800 Pleven, Bulgaria

**Keywords:** duplex ultrasound, renal artery stenosis, duplex ultrasound, cost, analysis, outcome

## Abstract

**Introduction:** Renal artery stenosis can significantly impact long-term graft survival rates following kidney transplant. Early recognition and management can improve the longevity of the kidney allograft. We aimed to evaluate the clinical role of duplex ultrasound in the diagnosis of renal artery stenosis (RAS). We also wanted to evaluate the current incidence of renal artery stenosis at our institute. **Methods:** A retrospective, consecutive series of 367 patients who underwent renal transplantation between 1 January 2020 and 30 December 2024 was conducted. We collected data regarding the recipients’ age, body mass index, and comorbidities. All patients diagnosed with renal artery stenosis were identified. The incidence of kidney transplant artery stenosis and presentation were recorded. All general physical parameters and laboratory data were collected and analyzed. **Results:** A total of 28 patients had initial suspicion of renal artery stenosis, documented via initial dedicated duplex ultrasound of the transplanted kidney. The initial mean systolic BP at initial US was 151 (99–213) mmHg, and mean creatinine was 2.43 (1.28–6.38) mg/dL. However, on repeat duplex ultrasound, three patients showed no features of renal artery stenosis and had no physical parameters consistent with RAS. A total of 25 patients diagnosed with RAS on initial duplex ultrasound underwent angiography. Twenty-four patients were confirmed with RAS on angiography, while one patient had a normal angiogram. Among patients diagnosed with TRAS, the mean resistive index was 0.71 ± 0.17 at the upper pole, 0.73 ± 0.19 at the mid pole, and 0.71 ± 0.21 at the lower pole. The mean peak systolic velocity was 462.57 ± 166.28 cm/s. **Conclusions:** Duplex ultrasound is an important initial tool for diagnosing transplant renal artery stenosis. An increase in peak systolic velocity was observed in our cohort; however, resistive indices were largely within acceptable limits. Management should be guided by clinical parameters (e.g., elevated systolic BP and rising creatinine) alongside imaging findings.

## 1. Introduction

Transplant renal artery stenosis (TRAS) is one of the most common and clinically significant pathologies [1]. It mostly occurs within 3 to 6 months following transplantation [2]. TRAS can also develop up to two years post-transplant. Late presentation is typically associated with progressive atherosclerotic disease [3]. Other vascular pathologies in renal transplantation include renal artery and renal vein thrombosis and pseudoaneurysms [1].

The clinical presentation of TRAS can range from asymptomatic cases to the development of hypertension and flash pulmonary edema [4,5]. The underlying stenosis can significantly reduce blood flow to the allograft, resulting in persistent ischemia. This compromised perfusion may lead to graft dysfunction and, if left untreated, can ultimately cause irreversible graft injury or loss. Early recognition is essential to assess the extent of damage and to salvage the graft.

TRAS can be detected using either noninvasive or invasive imaging techniques. Noninvasive methods include duplex ultrasound, CT angiography, and MR angiography, while invasive imaging primarily involves digital subtraction angiography (DSA). Duplex ultrasound is typically the initial modality used to assess the presence of TRAS. Measurements such as acceleration time, resistance index (RI), peak systolic velocity (PSV), and the PSV ratio within the renal vasculature help guide further clinical decision making. In the study by Fananapazir et al., the mean RI was 0.67 in patients with mild TRAS and 0.63 in those with severe TRAS, while the mean PSV was 431 cm/s and 459 cm/s, respectively [6]. However, TRAS can still occur in patients with normal or near-normal RI and PSV values, which makes the diagnosis particularly challenging [7,8]. In such cases, a definitive diagnosis can be made using DSA, which provides radiographic visualization of the vessels [9].

In this case series, we report on patients at our transplant center who developed TRAS following kidney transplantation. TRAS was identified using duplex ultrasonography. We describe the clinical characteristics of both donors and recipients and outline the clinical summary of TRAS in affected patients. We have reported the ultrasound parameters observed in our cohort, which will contribute to the growing body of literature on this important post-transplant complication. This article will assess the role and utility of performing early imaging investigations when an increase in creatinine or persistent hypertension is noted despite anti-hypertensive medication in kidney allograft recipients.

## 2. Methods and Materials

This retrospective case series was conducted following approval from the Institutional Review Board. Data from 367 renal allograft recipients who underwent transplantation at our center between 1 January 2020 and 30 December 2024 were reviewed.

Inclusion criteria consisted of all patients who underwent Duplex ultrasound due to clinical suspicion of TRAS post-transplantation. A standardized technique was used in all transplants, involving single renal artery anastomosis with a donor aortic patch to the external iliac artery. Exclusion criteria included pediatric recipients (<18 years of age) and patients without the clinical features of TRAS.

Electronic medical records were reviewed to collect recipient data, including demographic information (age, body mass index, race, and comorbidities) and clinical status at the time of TRAS diagnosis. Donor characteristics were also extracted. Imaging data, which included duplex ultrasound findings such as RI, PSV, and the anatomical location of the stenosis, were collected.

Initial clinical suspicion of TRAS was based on the following institutional criteria:

- Elevated serum creatinine (greater than or equal to 1.3 mg/dL);
- More than 50% renal artery stenosis on initial Duplex ultrasonography;
- Hypertension requiring two or more antihypertensive medications.

Patients with a functioning allograft who continued to require two or more antihypertensive agents post-transplant and had no clinical indication for dialysis were included in the study. These medications were either continued from their pre-transplant regimen or newly initiated after transplantation. Although a serum creatinine ≥1.3 mg/dL was part of the institutional criteria for suspecting TRAS, this parameter was interpreted in conjunction with clinical (hypertension) and radiological (duplex ultrasonography) findings to avoid misclassification, particularly in cases where elevated creatinine may reflect baseline donor characteristics, such as in deceased donor grafts.

All duplex ultrasound examinations were performed as part of routine post-transplant surveillance by board-certified radiologists using a Logiq E9, GE Healthcare (Chicago, IL, USA) ultrasound system, equipped with a linear transducer (3–8 MHz). The same model of ultrasound equipment was used for all subjects.

The analysis of data was performed using the Statistical Package for the Social Sciences (SPSS), Version 28 (IBM Corp, Armonk, NY, USA). Normality of continuous variables was assessed using the Shapiro–Wilk test. RI values measured at the upper, mid, and lower poles of the kidney were not normally distributed (*p* < 0.05), while PSV was normally distributed (*p* = 0.23). The Friedman nonparametric test was applied to compare RIs across different poles. A *p*-value ≤ 0.05 was considered statistically significant.

## 3. Results

Between 1 January 2020 and 30 December 2024, a total of 367 patients underwent renal transplantation at our center. Among these, 28 patients were identified with imaging findings suspicious for TRAS and were included in this study. The majority of patients in this case series were male, of African American ethnicity, and had a history of hypertension. All donors in this study were deceased donors. Of the deceased donors, four had a history of hypertension, and none had diabetes mellitus. Donor characteristics and detailed clinical profiles of the recipients are summarized in Table 1 and Table 2, respectively.

The average time from transplantation to presentation with suspected TRAS was 4.9 months (range: 0.4–17 months). At the time of presentation, the mean systolic blood pressure was 151 mmHg (range: 99–213 mmHg), and all patients had elevated serum creatinine levels with a mean of 2.43 mg/dL (range: 1.28–6.38 mg/dL). On average, patients were taking three antihypertensive medications (range: 2–5), as outlined in Table 3.

All 28 patients underwent duplex ultrasonography, which demonstrated abnormal vascular parameters. The individual duplex findings for all suspected TRAS cases, including RI values across poles, PSV measurements, and the anatomical location of the stenosis, are presented in Table 4. The mean RI was 0.71 ± 0.17 at the upper pole, 0.73 ± 0.19 at the mid-pole, and 0.71 ± 0.21 at the lower pole. The mean PSV was 462.57 ± 166.28 cm/s. A comparative analysis of RI values across the upper, middle, and lower poles and their relation with PSV is presented in Table 5. A Friedman test was conducted to compare the RI across the upper, mid, and lower poles in patients diagnosed with TRAS. The differences were not statistically significant, χ^2^(2) = 2.17, *p* = 0.338, indicating that RI was similar across poles.

Out of the 28 patients, 25 underwent digital subtraction angiography (DSA) for further evaluation. Case 25 showed no evidence of TRAS on DSA despite initial duplex findings. Cases 26 to 28 initially had features of TRAS on duplex imaging. However, these three cases did not demonstrate elevated BP (systolic < 140 mm of Hg), their creatinine levels were near normal, and they maintained steady serum creatinine levels on follow-up. Repeat duplex imaging within three months confirmed the resolution of findings in these patients. Based on confirmed imaging and clinical correlation, the frequency of TRAS in our cohort was determined to be 6.8%.

**Figure 1:**
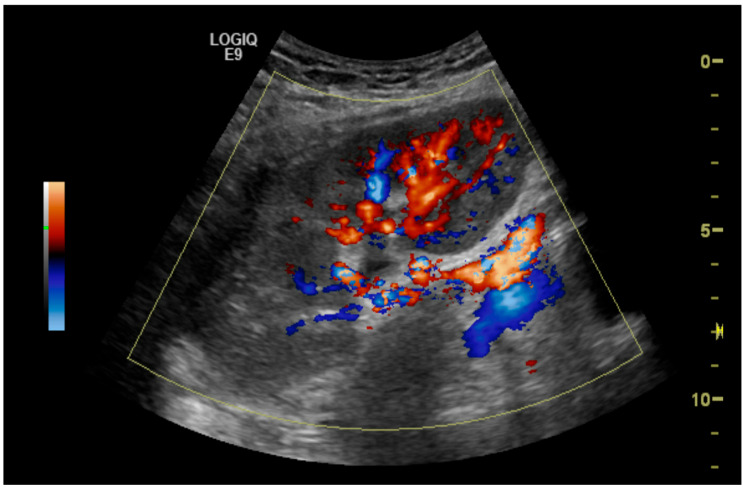
Renal artery stenosis (90%) at the site of anastomosis of the renal artery to the external iliac artery.

**Figure 2:**
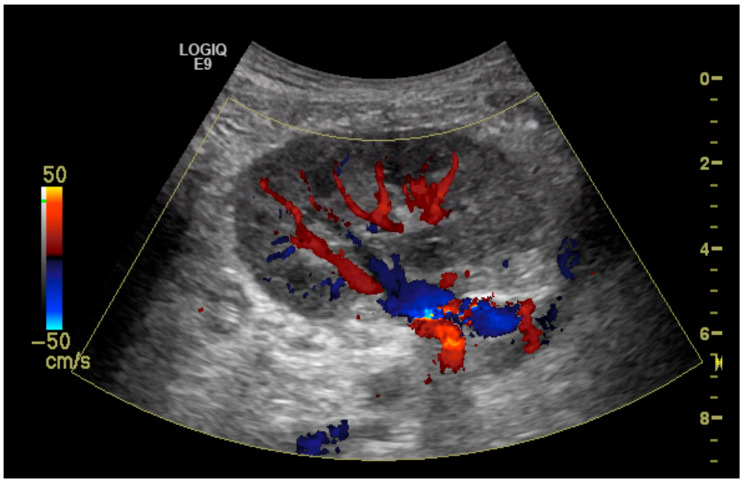
Renal artery stenosis (70%) demonstrated at the proximal part of the renal artery just above the anastomosis to the external iliac artery.

**Figure 3:**
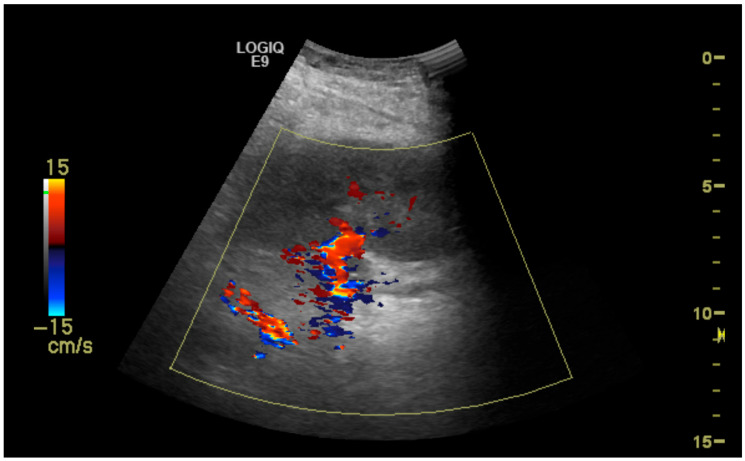
Stenosis (50%) at the midpoint of the transplanted renal artery.

**Figure 4:**
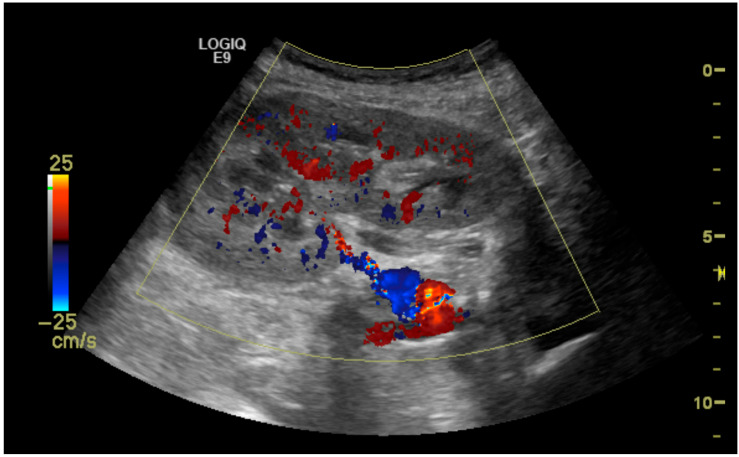
Stenosis (60%) of the renal artery at the hilum of the transplanted kidney.

The initial suspicion of TRAS was identified at the site of arterial anastomosis in 11 subjects (39%), proximally in 11 subjects (39%), at the mid-segment in 4 subjects (14%), and at the hilum in 2 subjects (7%). Representative cases are illustrated in Figure 1, Figure 2, Figure 3 and Figure 4. Figure 1 (Case 5) shows anastomotic narrowing with a PSV of 853 cm/s and 90% stenosis confirmed on DSA. Figure 2 (Case 4) demonstrates proximal renal artery stenosis. Figure 3 (Case 11) illustrates markedly elevated intrarenal resistance with an RI of 1.0 at the mid-point of the renal artery. Figure 4 (Case 14) depicts hilar stenosis with a PSV of 604 cm/s.

## 4. Discussion

The prevalence of TRAS is approximately 6%, while the incidence rate in the United States, based on Medicare insurance claims, is 8.3 cases per 1000 patient-years [10,11]. Reports from multiple centers have shown incidence rates ranging from 1% to 23%, which aligns with the findings at our institute [12]. The overall confirmed rate of TRAS in our case series was 6.5% which is in line with the literature. The occurrence of TRAS within 4.9 months post-transplantation is also consistent with previous studies [2].

The development of TRAS is influenced by both recipient- and donor-related factors. Several donor characteristics have been associated with an increased risk of TRAS, including advanced donor age, prolonged cold ischemia time, and elevated body mass index (BMI) [11,13]. Donor age over 40 years has also been associated with a higher incidence of TRAS. In our study, the mean deceased donor age was 36 years (range: 6 to 60). In addition, a cold ischemia time exceeding 26 h has been reported as a contributing factor, and the mean cold ischemia time in our series was 23 h (range: 12.5–40.2) [11]. Furthermore, an elevated donor BMI was implicated in the development of TRAS in a study by Wang et al. [13]. The kidney donor profile index (KDPI) is a percentage score based on donor characteristics categorized on a scale of 1 to 100%. A lower KDPI score suggests a kidney with more favorable characteristics and a higher expected longevity after transplant, while a higher KDPI score is associated with increased risk of graft failure and shorter expected organ survival [14].

Recipient baseline factors associated with the development of TRAS include the presence of comorbidities such as diabetes mellitus, hypertension, and pre-existing nephropathy. Both HTN (57%) and DM (32%) were common in our series of patients and were the cause of end-stage renal disease. In a study conducted by Kanhouche et al., who analyzed 274 suspected cases of TRAS, it was observed that TRAS was more common among older recipients (those with a mean age of 46.3 years) [4]. The mean age in our case series was 55.8 years (range: 27 to 71), higher than published series [7]. It is possible that age-related vascular changes, including pre-existing arteriolar pathology and oxidative stress, could play a role in contributing to the development of TRAS [15]. Hypertension and diabetes mellitus have also been associated with an increased incidence of TRAS in multiple studies [4,11,16]. Diabetes may contribute to TRAS by glycosylation of extracellular matrix proteins and inducing changes that impair the antiatherogenic properties of vasculature, which could lead to vascular endothelial dysfunction [17]. Similarly, hypertension can also contribute to vascular inflammation and remodeling, both of which may play a role in the pathogenesis of TRAS [18]. Pre-existing recipient nephropathies, as seen in some of our cases, may also influence the prognosis of transplantation [19]. The American Society of Anesthesiologists (ASA) score was used at our center for assessment of kidney recipients’ peri-operative risk assessment (Table 2). This assessment guided the intra-operative and post-operative course. All patients with an ASA score of 4 were admitted post-operatively to intensive care for a minimum of 24 h to monitor initial management.

Patients with TRAS at the time of the initial radiographic assessment have generally been reported to have a serum creatinine level around 2.1 mg/dL, a systolic blood pressure of greater than 150 mmHg, and a time interval of almost 6 months from transplantation [4]. Our findings are similar to this clinical profile, suggesting that these parameters may frequently be present at the time of TRAS presentation. Early clinical presentation of TRAS includes elevated blood pressure and increased serum creatinine. Early detection of this pattern could facilitate timely evaluation and intervention, potentially improving graft outcomes.

Surgical technique has also been identified as a potential contributor to the etiopathogenesis of TRAS. The absence of an aortic patch (live donor allograft), technical variations in suture materials, and use of the cross-clamping technique can all contribute to injury of the renal artery [20]. Further, cases requiring vascular reconstruction of multiple renal arteries have been associated with a higher incidence of TRAS [21]. In a study by Choudhary et al., the occurrence of TRAS was found to be more than three times higher in patients with multiple arterial reconstructions associated with an earlier onset of transplant [21]. Post-transplant hemodynamic changes may further contribute by inducing oscillatory shear stress, which can induce oxidative stress and intimal hyperplasia [22]. Furthermore, in proinflammatory states, vascular smooth muscle cells may transition from a contractile to a synthetic phenotype, a shift thought to play a role in vascular remodeling and dysfunction [23].

Duplex ultrasonography is the most commonly used screening tool in clinical practice for detecting TRAS. It has a sensitivity greater than 90% and a specificity of greater than 80% for diagnosing TRAS [6]. However, its accuracy is highly dependent on the skill and experience of the operator [12]. In addition to identifying the presence and location of stenosis, duplex ultrasonography provides information to assess the severity of the lesion through hemodynamic measurements such as PSVs and RIs.

In a recent meta-analysis by Pini et al., who analyzed data on TRAS from 2000 to 2020, the anastomotic (para-anastomotic) site was identified as the most frequent location of stenosis [2]. Similarly, in a study from India, Muske et al. reported that stenosis at the site of anastomosis was present in half of their patient population [24]. Our findings are consistent with these reports, as we observed that 39% of cases involved the arterial anastomosis. This pattern may be attributed to increased susceptibility of the anastomotic region to stenosis, potentially related to surgical technique, localized vascular trauma during the procedure, and tissue remodeling during the healing process. These factors likely contribute to the higher prevalence of stenosis observed in this area across different patient populations and settings.

PSVs and RIs are two hemodynamic parameters commonly used in the evaluation of TRAS. PSV measures the maximum blood flow velocity within the renal artery during systole. Normal PSV in transplanted renal arteries is <150 cm/s [25]. In a study by Qi et al., the mean pretreatment PSV in patients with TRAS was 339 cm/s [26]. This is comparable to the mean PSV of 462 cm/s seen in our patient group. Although the absolute values differ, they fall within the expected pathological range for significant stenosis, thereby supporting the comparability in clinical interpretation.

In a study by Baxter et al., a PSV of ≥250 cm/s demonstrated a sensitivity of 100% and a specificity of 95% for detecting TRAS [27]. In contrast, Qi et al. reported that a PSV > 257.5 cm/s had a sensitivity of 78.57% and a specificity of 100% [26]. These discrepancies in reported sensitivity and specificity may be due to differences in methodology, patient demographics, and thresholds for defining stenosis severity. Despite these variations, the consistently high sensitivity and specificity highlight PSV as a valuable diagnostic tool. In the present study, we did not calculate sensitivity or specificity, as it was a descriptive case series without a control group, making such analysis not applicable.

RI is used to measure intrarenal vascular resistance using the following formula: (peak systolic velocity − end diastolic velocity)/peak systolic velocity [28]. Normal RI values typically range below 0.7 to 0.8. Values greater than 0.8 are considered abnormal [29]. Elevated RI has been associated with poor graft outcomes and may be a potential early sign of the development of TRAS [30]. In our study, only 15 (54%) cases had RIs more than 0.7. All these patients had findings of TRAS on the duplex ultrasound imaging. However, its utility in routine clinical practice remains uncertain due to limited supporting evidence [28]. Furthermore, a tardus parvus waveform, associated with a slow rise in systolic upstroke and a delayed peak, can be associated with stenosis and reduced arterial compliance [31].

While duplex ultrasonography is useful for initially assessing hemodynamic parameters, it has limitations that can decrease the diagnostic accuracy [32]. Errors in probe angle correction can lead to underestimation of PSV, potentially causing a missed diagnosis [33]. In cases of severe post-stenotic TRAS, the RI may normalize, potentially masking the true severity of the disease [34]. These limitations highlight the need for digital subtraction angiography, the definitive diagnostic modality and gold standard [18].

In cases with minimal hypertension and only mild changes in hemodynamic parameters, treatment with angiotensin inhibitors or angiotensin receptor blockers can be initiated [12]. These patients also require close monitoring and repeated imaging for monitoring of hemodynamic parameters. If the patient’s serum creatinine level remains elevated and their systolic blood pressure, despite medical management, remains high, then radiological intervention must be pursued. In a study by Macchini et al., who compared percutaneous angioplasty (PTA) to angioplasty with stent placement for the treatment of TRAS, better postoperative clinical outcomes were seen in the stent group [35]. The reintervention rate was 30% in patients treated with PTA alone, compared to just 5% in those who received stents, regardless of preliminary PTA status.

Following successful therapeutic intervention for TRAS, a notable reduction in serum creatinine and systolic blood pressure is typically observed. This trend has been reported in previous studies, including those by Qi et al. and Macchini et al. [26,35]. These findings support the potential of timely therapeutic intervention to correct underlying dysfunction, prevent further deterioration of renal function, and, ultimately, reduce the risk of allograft loss. Timely treatment may also help to lower the long-term healthcare costs associated with graft dysfunction and failure.

This study has several limitations, including its retrospective design, lack of blinding, operator variability, absence of a control group, and confinement to a single center. The retrospective nature introduces potential biases, such as limited control over management protocols and the possibility of incomplete documentation of clinical data. Clinically asymptomatic patients were excluded, as the study focused on symptomatic TRAS cases. The radiologists’ awareness of clinical information may have contributed to observer bias. The single-center setting further restricts the generalizability of the findings. Future studies incorporating multiple centers and including a control group would improve the validity and clinical relevance of the results.

## 5. Conclusions

Duplex ultrasound remains a valuable diagnostic tool in the evaluation of transplant renal artery stenosis. In our cohort, elevated peak systolic velocities were noted, while resistive indices generally remained within acceptable ranges. Clinical management should be individualized, guided by a combination of imaging findings and clinical indicators such as rising systolic blood pressure and deteriorating renal function. Confirming the diagnosis will require angiography.

## Figures and Tables

**Table 1:** Donor characteristics, including age, KDPI (KDPI: kidney donor profile index), and cold ischemia time.

	Mean (Range)
**Age (years)**	36 (6–60)
**KDPI score**	41 (2–90)
**Cold ischemia time (hours)**	23 (12.5–40.2)

**Table 2:** Clinical characteristics of recipients diagnosed with TRAS. American Society of Anesthesiologists score (ASA score).

	Frequency (*n*)	Percentage (%)
**Mean age** (years)	55.8 (27 to 71)	-
**Body mass index** (kg/m^2^)	29.51 (20 to 37.9)	-
**Gender**		
Male	17	60.7
Female	11	39.3
**Race**		
African American	21	75
Caucasian	5	18
Hispanic	2	7
**Comorbidity**		
Hypertension	16	57
Diabetes mellitus	9	32
Interstitial nephritis	2	7
Autoimmune nephropathies	1	4
**ASA score**		
3	23	82
4	5	18

**Table 3:** Recipient characteristics at the time of RAS diagnosis. RAS: renal artery stenosis; BP: blood pressure.

	Mean (Range)
**Time from transplant to RAS diagnosis (months)**	4.9 (0.4–17)
**BP systolic (mmHg)**	151 (99–213)
**BP diastolic (mmHg)**	76 (55–120)
**Creatinine (mg/dL)**	2.43 (1.28–6.38)
**Prescribed antihypertensive medications**	3 (2–5)

**Table 4:** Resistive indices, peak systolic velocity, and stenosis location of suspected stenosis.

Case No.	Resistive Indices			Peak Systolic Velocity (cm/s)	Location
	Upper	Mid	Lower		
**1**	0.5	0.5	0.5	548	Anastomosis
**2**	0.68	0.67	0.67	434	Anastomosis
**3**	0.6	0.5	0.5	669	Anastomosis
**4**	0.73	0.67	0.69	494	Proximal
**5**	0.45	0.43	0.47	535	Anastomosis
**6**	0.7	0.7	0.7	420	Proximal
**7**	0.63	0.69	0.59	679	Proximal
**8**	1	1	0.82	450	Proximal
**9**	0.58	0.63	0.54	348	Anastomosis
**10**	0.71	0.69	0.7	228	Anastomosis
**11**	1	1	1	293	Mid
**12**	0.76	0.78	0.74	324	Proximal
**13**	0.74	0.73	0.64	462	Anastomosis
**14**	0.67	0.7	1	604	Hilum
**15**	1	1	1	544	Anastomosis
**16**	0.59	0.56	0.43	319	Mid
**17**	1	1	1	853	Anastomosis
**18**	0.83	0.86	0.77	481	Anastomosis
**19**	0.43	0.57	0.61	713	Proximal
**20**	0.77	0.78	1	465	Anastomosis
**21**	0.75	0.7	0.77	403	Proximal
**22**	0.68	1	1	770	Mid
**23**	0.54	0.36	0.29	450	Hilum
**24**	0.7	0.77	0.73	304	Proximal
**25**	1	1	1	285	Proximal
**26**	0.49	0.63	0.62	234	Proximal
**27**	0.70	1.0	0.61	235	Mid
**28**	0.58	0.57	0.43	408	Proximal

**Table 5:** Resistive index (RI) values are compared across the upper, middle, and lower poles of the kidney. Peak systolic velocity (PSV) is presented descriptively. The *p*-value indicates no statistically significant difference in RI among the three poles.

		Mean	Standard Deviation	*p*-Value	95% Confidence Interval
Resistive Index	Upper pole	0.71	0.17	0.338	0.64–0.77
	Mid Pole	0.73	0.19		0.66–0.80
	Lower Pole	0.71	0.21		0.63–0.79
Peak systolic velocity (cm/s)		462.57	166.28	-	400.98–524.16

## Data Availability

All data supporting the findings of this case series are included within the manuscript.

## Abbreviations

ACEIs	Angiotensin-converting enzyme inhibitors
ARBs	angiotensin receptor blockers
BMI	body mass index
DSA	digital subtraction angiography
ESRD	end-stage renal disease
IR	interventional radiology
PSV	peak systolic velocity
PTA	percutaneous transluminal angioplasty
RI	resistance index
SPSS	Statistical Package for the Social Sciences
TRAS	transplant renal artery stenosis
